# Cerebroretinal Microangiopathy with Calcifications and Cysts (CRMCC): A 5-Year Diagnostic Challenge

**DOI:** 10.3390/diagnostics16101432

**Published:** 2026-05-08

**Authors:** Mikayla J. Foley, Michael Cole, Carolina Sandoval-Garcia, Robert T. Galvin, William B. Dobyns, Collin M. McClelland, Can Özütemiz

**Affiliations:** 1 Department of Radiology, University of Minnesota, Minneapolis, MN 55455, USA; 2 Department of Neurology, University of Minnesota, Minneapolis, MN 55455, USA; 3 Department of Neurosurgery, University of Minnesota, Minneapolis, MN 55455, USA; 4 Department of Pediatrics (Hematology/Oncology), University of Minnesota, Minneapolis, MN 55455, USA; 5 Department of Pediatrics (Genetics), University of Minnesota, Minneapolis, MN 55455, USA; 6 Department of Ophthalmology & Visual Neurosciences, University of Minnesota, Minneapolis, MN 55455, USA

**Keywords:** CRMCC, Coats-plus, CTC1, calcifications, intracranial cysts, 7 Tesla, magnetic resonance imaging

## Abstract

**Background and Clinical Significance**: CTC1-related cerebroretinal microangiopathy with calcifications and cysts (CRMCC), or Coats-plus syndrome, is an extremely rare autosomal recessive telomere-dysfunction disorder. A total of 29 cases in 15 reports have been reported in the English literature. The primary imaging characteristics include leukoencephalopathy, intracranial calcifications, and parenchymal cysts. **Case Presentation**: We describe a patient with CRMCC, who presented with a large intracranial cystic mass and basal ganglia calcifications, with imaging findings strongly mimicking a primary CNS tumor. The patient underwent multiple surgeries with inconclusive biopsies. Ultimately, it took five years and the collaboration of several specialists to arrive at the final diagnosis. Furthermore, we present dedicated clinical 7T orbit MRI findings on the patient’s brother, who has the same disease. **Conclusions**: We present a rare case of CRMCC with lack of overt leukoencephalopathy at presentation and absence of characteristic extracranial/extraocular manifestations, significantly complicating diagnosis. Furthermore, to the best of our knowledge, we share the first reported clinical 7T orbital MRI in the pediatric population.

## 1. Introduction

CTC1-related cerebroretinal microangiopathy with calcifications and cysts (CRMCC), or Coats-plus syndrome, is an extremely rare autosomal recessive disorder. It is characterized by the neuroimaging triad of intracranial calcifications, intracranial parenchymal cysts, and leukoencephalopathy, plus retinal findings such as telangiectasias, vitreous hemorrhages, and subretinal exudates [1,2]. This report describes imaging findings in a 9-year-old girl with CRMCC who presented with a large posterior fossa cystic mass mimicking a primary CNS neoplasm without overt leukoencephalopathy. The patient remained undiagnosed for five years until Coats disease was identified in her younger brother, suggesting a genetic etiology. We also present the brother’s clinical 7T orbital MRI findings. To the best of our knowledge, pediatric clinical 7T orbital MRI images have not been previously published, and no CRMCC case has been published in a radiology-focused journal, underscoring the significance of this unique case.

## 2. Case Report

**Figure 1:**
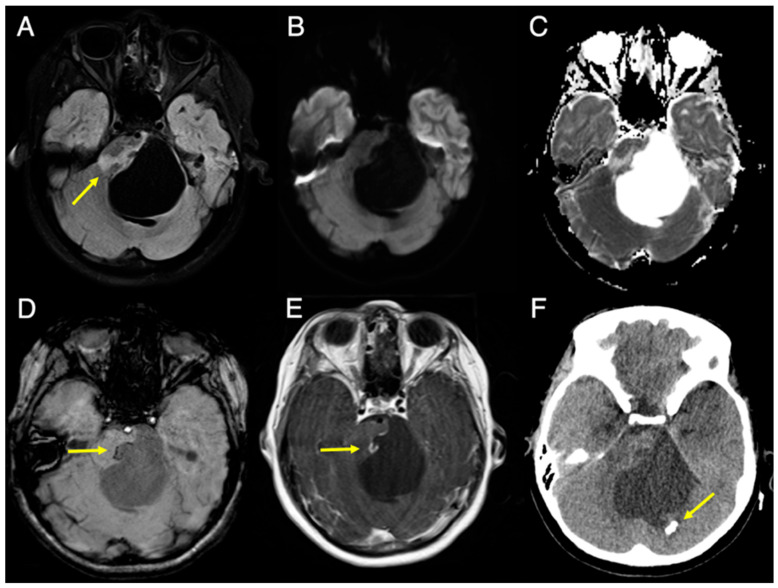
Axial 3T MRI and CT slices of the cystic lesion at the level of the pons. (**A**) Fat-saturated FLAIR image shows patchy T2 hyperintensity in the adjacent pons (arrow). (**B**) DWI and (**C**) ADC map shows facilitated diffusion inside the cyst. (**D**) SWI shows linear focus of susceptibility, possibly representing microhemorrhage or calcification in the cyst/pontine parenchyma junction (arrow). (**E**) Contrast-enhanced T1-TSE shows corresponding linear enhancement (arrow). (**F**) Same-day CT image (brain window) shows a coarse calcification in the posterior cyst wall (arrow).

**Figure 2:**
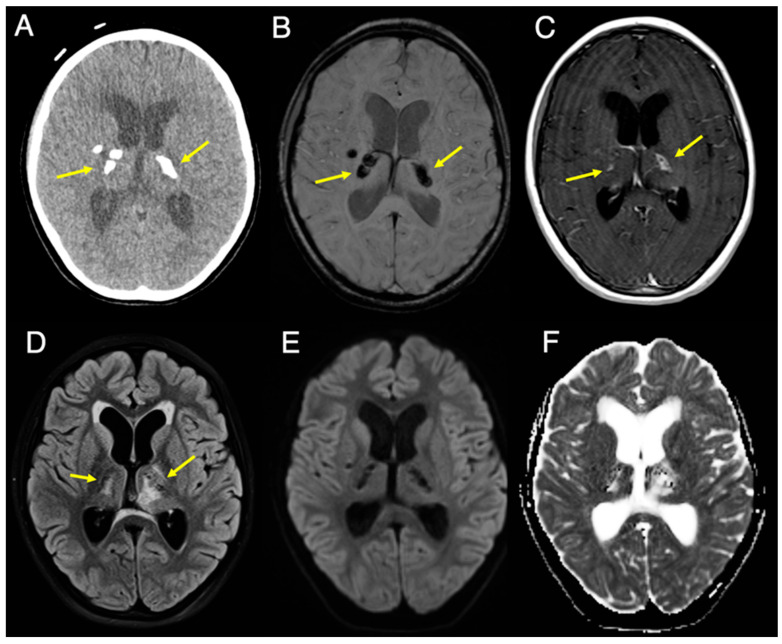
Axial CT and 3T MRI slices at the level of the basal ganglia. (**A**) Same-day CT image (brain window) and (**B**) SWI image shows bilateral coarse basal ganglia calcifications, primarily involving the thalami (arrows). (**C**) Contrast-enhanced T1-TSE image shows mild peripheral enhancement surrounding the calcifications (arrows). (**D**) Fat-saturated FLAIR image shows peripheral T2 hyperintensities (arrows) surrounding the calcifications, similar to those seen in the pons in Figure 1. (**E**,**F**) DWI and ADC map show no associated diffusion restriction.

A previously healthy 9-year-old girl presented to her pediatrician with a 3-month history of ataxia and left eye blurriness. Physical examination revealed mild facial asymmetry, left facial weakness, horizontal lateral gaze nystagmus, rightward tongue deviation, and ataxia. An urgent brain MRI and head CT (Figure 1 and Figure 2) were obtained. 3T MRI revealed a 4.9 × 5.0 cm cystic lesion, likely arising from the pons (Figure 1). The cyst was causing significant mass effect on the fourth ventricle with associated obstructive hydrocephalus (Figure 2) and compression of the left 7th/8th cranial nerve complex. The cyst content was entirely suppressed on FLAIR without diffusion restriction. Patchy T2 hyperintensity with a T2-shine-through effect was present in the adjacent pons, likely secondary to compression or leukoencephalopathy (Figure 1A–C). The cyst wall arising from the pons demonstrated a linear focus of susceptibility on SWI, suggesting either microhemorrhage or calcification (Figure 1D). Corresponding linear enhancement was seen on post-gadolinium T1-TSE sequences (Figure 1E). On CT, there was no corresponding calcification in the pons, but a coarse calcification was present in the posterior cyst wall (Figure 1F). In addition, bilateral coarse basal ganglia calcifications were observed, mainly involving the thalami (Figure 2A,B) with mild peripheral contrast enhancement (Figure 2C). These lesions were dark on FLAIR and DWI but exhibited peripheral T2 hyperintensity (Figure 2D–F).

The patient was immediately admitted and treated with dexamethasone, resulting in slight symptomatic improvement. Urgent surgical intervention with tissue sampling was recommended. During surgery, no abnormal tissue was found within the cyst wall, and the cyst content was viscous and avascular. Several samples of this transparent mucinous material were sent to pathology. Biopsy of the cyst wall was attempted but was limited due to changes in intraoperative neuromonitoring of cranial nerves VII and VIII. Postoperatively, her nystagmus and facial weakness improved.

Pathological exam revealed largely acellular proteinaceous content with clusters of glial cells, with no evidence of mitoses, necrosis, microvascular proliferation, or Rosenthal fibers. No evidence of tumor was identified. Extensive serum laboratory testing was performed, including cryptococcosis, echinococcosis, cysticercosis, tuberculosis, toxoplasmosis, and human immunodeficiency virus, all of which were unrevealing. The Karius Spectrum test (microbial metagenomic sequencing) did not detect any organisms at statistically significant levels. MRI of the entire spine was negative for additional cystic masses or leptomeningeal disease. During the postoperative ophthalmologic assessment, the patient was found to have acquired nystagmus likely related to the brainstem cystic lesion, which improved over time.

**Figure 3:**
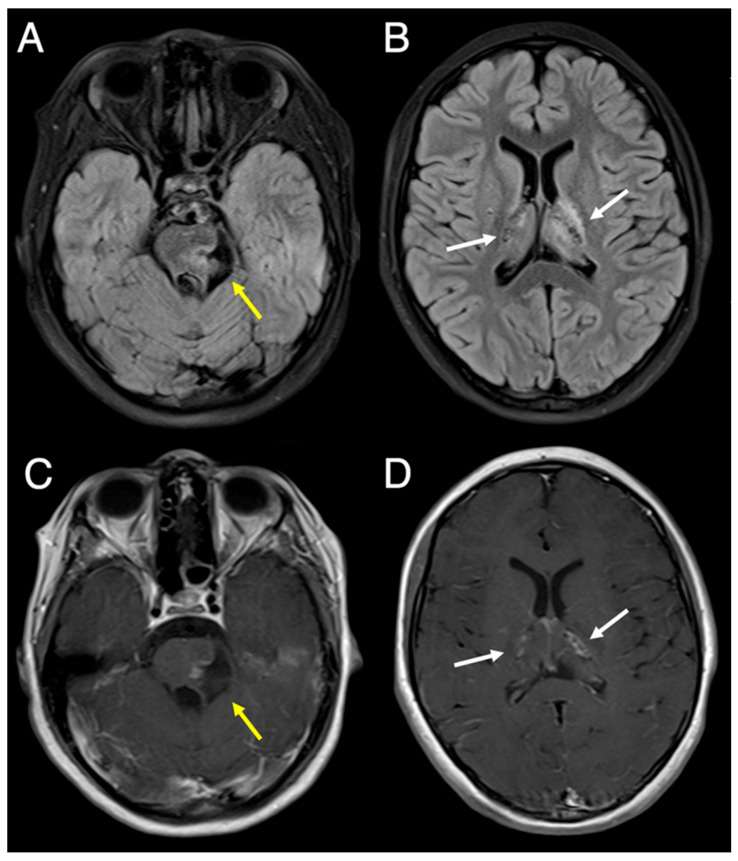
Axial 3T MRI slices one year after surgery at the level of the pons and basal ganglia, respectively. (**A**,**B**) Fat-saturated FLAIR images; (**C**,**D**) contrast-enhanced T1-TSE images show an appropriate degree of decompression of the brainstem cyst (yellow arrows) and stability of the basal ganglia lesions (white arrows).

**Figure 4:**
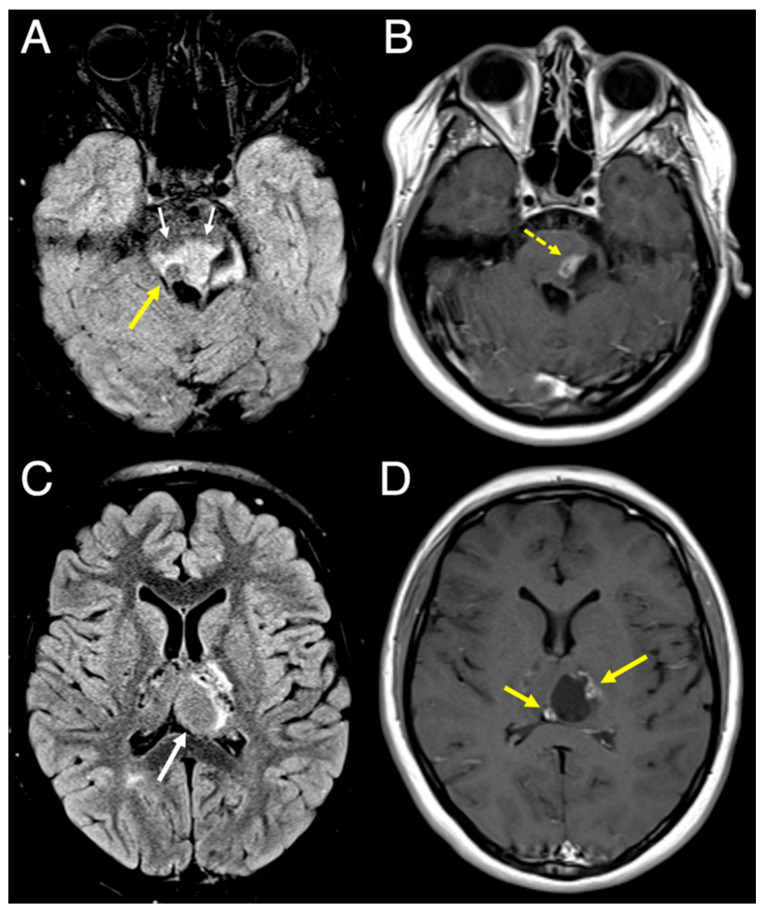
Five years after surgery, (**A**) fat-saturated FLAIR and (**B**) contrast-enhanced T1-TSE images show interval development of a new cyst (yellow arrow) in the right posterolateral aspect of the brainstem with surrounding abnormal white matter signal (white arrows) and worsening enhancement adjacent to prior residual cyst (dashed arrow) in the left aspect of the brainstem. (**C**) Fat-saturated FLAIR and (**D**) contrast-enhanced T1-TSE images at the basal ganglia level show new proteinaceous cyst formation (white arrow) in the left thalamus with surrounding linear and nodular enhancement (yellow arrows).

After surgical decompression, the patient appeared clinically improved. Unfortunately, pathology failed to provide a definitive diagnosis. The patient was ultimately followed for several years under a diagnosis of “cystic mass of unknown origin.” During that time, several follow-up brain MRIs documented stability of the basal ganglia lesions (Figure 3). Unfortunately, four years later, she developed new similar cysts and white matter lesions, particularly in the left thalamus with mass effect (Figure 4). Despite the involvement of multiple different specialties, a unified diagnosis was still not reached.

One day, five years after presentation, a neurologist proposed a genetic etiology. Specifically, they suspected “leukoencephalopathy with cerebral calcifications and cysts” (LCC), prompting a genetics referral. It was then discovered that the patient’s younger brother was recently diagnosed with Coats disease, further supporting a genetic etiology. The patient’s genetic testing revealed a pathologic variant in the CTC1 gene, establishing the diagnosis of CTC1-related CRMCC.

This unified diagnosis explained both the intracranial findings and ophthalmologic symptoms. Although MRI did not reveal evidence of Coats disease in the orbits, a fluorescein angiography later confirmed the presence of characteristic retinal telangiectatic vessels. 

**Figure 5:**
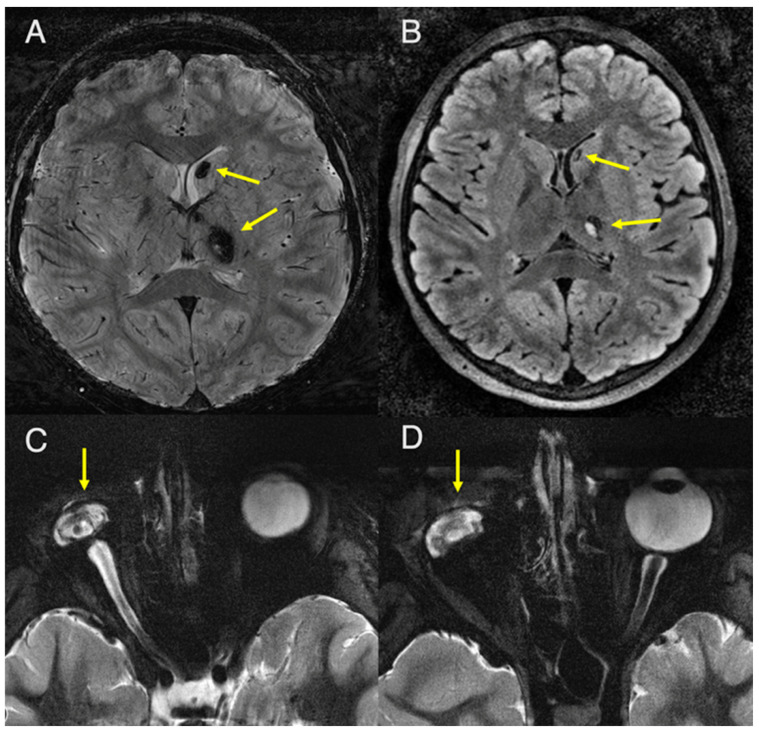
Axial 7T MRI slices obtained from the patient’s younger brother with Coats disease. (**A**) T2* image (TR/TE/Slice thickness: 1560 ms/20 ms/1.5 mm) and (**B**) 3D-FLAIR-SPACE image (TR/TE/Slice thickness: 7000 ms/396 ms/0.4 mm) of the whole brain show coarse basal ganglia calcifications, predominantly involving the thalami and left caudate nucleus (arrows). (**C**,**D**) Axial 7T T2 STIR images of the orbits (TR/TE/TI/Slice thickness: 6000 ms/67 ms/320 ms/2 mm) in subsequent axial slices show right microphthalmia, retinal detachment, intraocular proteinaceous debris, and intraocular blood products, representing chronic sequelae of Coats disease (arrows).

Genetic testing of the patient’s brother with Coats disease revealed the same CTC1 variant; thus, he was also referred for brain and orbital imaging. 7T-MRI showed similar parenchymal coarse calcifications in the basal ganglia, predominantly involving the thalami, left caudate nucleus, and cerebellum (Figure 5A,B). Adjacent to the left thalamic calcification, there was a similar mild T2-FLAIR signal and subtle peripheral enhancement. No cysts were visualized. 7T-MRI of the orbits revealed chronic sequelae of Coats disease, including right microphthalmia, retinal detachment, intraocular proteinaceous debris, and intraocular blood products (Figure 5C,D).

## 3. Discussion

CRMCC is an autosomal recessive telomere dysfunction disorder caused by mutations in the CTC1 gene on chromosome 17p13.1 [1,3]. CTC1-related CRMCC is extremely rare, with only 15 prior reports documenting a total of 29 cases reported in the English literature (Table 1) [4,5,6,7,8,9,10,11,12,13,14,15,16,17,18]. Various tissues express the CTC1 gene, including endothelial cells, and its pathogenesis is thought to involve small vessel obliterative microangiopathy [2,19,20]. For the intracranial findings, specifically, it is hypothesized that gradual obliterative angiopathy of small vessels leads to slow necrosis, cyst formation, and dystrophic calcifications [10]. On histopathology, telangiectatic vessels with thickened, hyalinized, calcified walls are typical [2,19]. In addition to intracranial findings, several multisystem manifestations have been described in CRMCC, including postnatal growth restriction, gastrointestinal bleeding, portal hypertension, skeletal abnormalities, osteopenia, fractures, and depigmented hair (Table 2) [1,2,5,11,21]. Of note, these extracranial manifestations were not observed in our patients.

Prior to diagnosis, a wide differential was considered. A primary pediatric CNS tumor was initially highest on the differential, particularly juvenile pilocytic astrocytoma (JPA) or ependymoma. However, less than 20% of JPAs calcify, and one would expect an enhancing nodular component [22]. Calcifications are more common in ependymomas compared to JPAs, although a solid nodular component and classic plastic-tumor appearance would be expected. In addition, basal ganglia calcifications are rare in these tumors. Infectious etiologies, including TORCH infections, were considered; however, the calcifications seen in TORCH infections are typically more periventricular or diffuse. Furthermore, TORCH infections are typically accompanied by brain malformations, such as microcephaly, macrocephaly, porencephaly, heterotopia, lissencephaly, or polymicrogyria [23]. Other infectious etiologies including tuberculosis were considered, but extensive laboratory work-up was unrevealing.

Ophthalmologic manifestations of CRMCC are like those of Coats disease, hence the eponym “Coats-plus” syndrome [1,3]. Like previously mentioned, the condition is classically characterized by the triad of leukoencephalopathy, intracranial calcifications (predominantly involving the basal ganglia), and parenchymal cysts on neuroimaging. While radiologic differentiation of LCC and CRMCC can be challenging [1,19], the leukoencephalopathy in LCC can be more diffuse or prominent compared to CRMCC. Furthermore, clinical features can help distinguish these two entities, as LCC is not typically associated with retinopathy or other extracranial manifestations [1,3,19]. Overall, genetic testing is required for definitive diagnosis of CRMCC. Management of this genetic condition is symptom-focused, primarily involving supportive care. For instance, treatments may include anticonvulsants for seizures, laser coagulation for retinal telangiectasias, or intracranial cyst decompression to decrease mass effect.

## 4. Conclusions

In this study, we describe a rare case of CRMCC without clear leukoencephalopathy at presentation, significantly complicating diagnosis. Furthermore, there was lack of extracranial manifestations—aside from retinal telangiectasias—which complicated the diagnostic process. Because of this, the patient remained undiagnosed for five years despite the involvement of several specialists. Ultimately, genetic analysis revealing a CTC1 mutation established the diagnosis of CRMCC. Furthermore, to the best of our knowledge, we share the first reported clinical 7T orbital MRI in the pediatric population.

On neuroimaging, CRMCC is characterized by the triad of leukoencephalopathy, intracranial calcifications, and parenchymal cysts. Specifically, review of the current literature suggests a single radiologic hallmark: basal ganglia calcifications (primarily thalamic) with peripheral enhancement along with parenchymal cysts. Limitations of the current report include small sample size, retrospective nature, and the lack of formal genetic methodology. As a future learning point, if a radiologist or trainee encounters such findings on a brain MRI, the rare genetic etiology of CTC1-related CRMCC should be considered in the differential, even in the absence of typical leukoencephalopathy or clear extracranial manifestations. In such cases, ophthalmologic and genetic consultation is recommended.

## Figures and Tables

**Table 1:** Reported CRMCC Cases in the English Literature with Confirmed CTC1 Mutations and Documented Imaging Findings.

Source	Number of Cases	Patient Demographics (Age at Onset, Sex)	Retinal Findings	Cerebral Calcifications	Leukoencephalopathy	Intracranial Cysts
Collin (2019) [6]	1	0, Unspecified	+	+	+	+
Liang (2021) [7]	1	3 months, M	+	−	+	−
López-Cañizares (2022) [8]	2 *	0, M	+	+	−	−
		0, M	+	+	−	−
Xu (2017) [4]	1	12 years, F	+	+	+	+
Gowda (2026) [9]	1	Unspecified, M	−	+	+	+
Romaniello (2013) [10]	1	7 months, M	+	+	+	+
Polvi (2012) [11]	14 **	6 months, F	+	+	+	+
		11 months, F	+	+	+	+
		18 months, F	+	+	+	+
		6 months, F	+	+	+	−
		7 months, F	+	+	+	−
		6 months, F	+	+	+	+
		0, M	+	+	+	+
		14 years, M	−	+	+	−
		1 month, M	+	+	+	−
		1 month, M	+	+	+	−
		1 year, F	+	+	+	−
		5 years, M	+	+	+	−
		0, M	+	+	+	+
		11 months, M	+	+	+	+
Mansukhani (2017) [1]	1	8 months, F	+	+	+	+
Netravathi (2015) [12]	1	18 months, M	+	+	+	+
Kayarian (2023) [13]	1	38 years, F	+	+	−	−
Sears (2022) [14]	1	0, F	+	+	+	−
Serrão (2025) [15]	1	46 years, F	+	+	+	−
Bozkurt (2022) [16]	1	6 months, F	+	+	−	+
Lin (2017) [17]	1	12 years, F	+	+	+	+
Chaaya (2025) [18]	1	23 years, F	+	+	−	−

* Patients are siblings. ** Data from 4 unrelated individuals with 11 additional affected family members, from 10 families.

**Table 2:** Characteristic Clinical and Radiological Findings of CRMCC.

Neuroimaging Findings	Clinical Findings
Basal ganglia calcifications (especially thalamic)	Postnatal growth restriction
Leukoencephalopathy	Gastrointestinal bleeding
Parenchymal cysts	Portal hypertension
	Skeletal abnormalities
	Osteopenia
	Fractures
	Depigmented hair

## Data Availability

The data presented in this study are contained within the article. Additional information may be available from the corresponding author upon reasonable request, subject to ethical and privacy restrictions.

## Abbreviations

The following abbreviations are used in this manuscript:

CRMCC	Cerebroretinal microangiopathy with calcifications and cysts
LCC	Leukoencephalopathy with cerebral calcifications and cysts
JPA	Juvenile pilocytic astrocytoma
